# A Rapid Digital PCR System with a Pressurized Thermal Cycler

**DOI:** 10.3390/mi12121562

**Published:** 2021-12-15

**Authors:** Xuee Chen, Qi Song, Beini Zhang, Yibo Gao, Kai Lou, Yiteng Liu, Weijia Wen

**Affiliations:** 1 Department of Physics, The Hong Kong University of Science and Technology, Clear Water Bay, Kowloon, Hong Kong; xchendi@connect.ust.hk (X.C.); qisong@ust.hk (Q.S.); 2 Guangzhou HKUST Fok Ying Tung Research Institute, Guangzhou 511458, China; 3 Advanced Materials Thrust, Department of Physics, The Hong Kong University of Science and Technology, Clear Water Bay, Kowloon, Hong Kong; bzhangay@connect.ust.hk; 4 Zhuhai Shineway Biotech Co., Ltd., Zhuhai 519000, China; gyb@swtech.me; 5 Guangzhou Kayja-Optics Technology Co., Ltd., Guangzhou 511458, China; louklijun@gmail.com; 6 Earth, Ocean and Atmospheric Sciences Thrust, Department of Physics, The Hong Kong University of Science and Technology, Clear Water Bay, Kowloon, Hong Kong; yliudx@connect.ust.hk

**Keywords:** digital PCR, static droplets array (SDA), high-pressure thermal cycler

## Abstract

We designed a silicon-based fast-generated static droplets array (SDA) chip and developed a rapid digital polymerase chain reaction (dPCR) detection platform that is easy to load samples for fluorescence monitoring. By using the direct scraping method for sample loading, a droplet array of 2704 microwells with each volume of about 0.785 nL can be easily realized. It was determined that the sample loading time was less than 10 s with very simple and efficient characteristics. In this platform, a pressurized thermal cycling device was first used to solve the evaporation problem usually encountered for dPCR experiments, which is critical to ensuring the successful amplification of templates at the nanoliter scale. We used a gradient dilution of the hepatitis B virus (HBV) plasmid as the target DNA for a dPCR reaction to test the feasibility of the dPCR chip. Our experimental results demonstrated that the dPCR chip could be used to quantitatively detect DNA molecules. Furthermore, the platform can measure the fluorescence intensity in real-time. To test the accuracy of the digital PCR system, we chose three-channel silicon-based chips to operate real-time fluorescent PCR experiments on this platform.

## 1. Introduction

Digital PCR technology, as opposed to real-time fluorescence quantitative PCR, is an absolute quantitative nucleic acid amplification technology that can detect DNA or RNA samples with very low copy numbers, greatly improving detection sensitivity [1,2]. The fabrication bottleneck of digital PCR has been broken with the development of microfluidic technology and micro-electro-mechanical systems (MEMS), which has greatly accelerated the development of digital PCR. Digital PCR has recently emerged as an emerging technology for biomolecule detection, capable of single-molecule counting and absolute quantification. It has broad application prospects in many fields such as rare gene mutation detection [3,4], copy number variation detection [5,6], gene sequencing [7], tumor marker detection [8,9], food safety [10], and environmental detection [11].

Performing PCR initiation with one target molecule is a signal amplification-based detection strategy in which the signal can be exponentially accumulated during digital PCR amplification. The concentration of a single molecule must be increased to the detection limit of the detector before single-molecule detection can be realized. Given that the analyst is a single molecule, reducing the reaction volume is an effective way to increase the analyst concentration. However, because the reaction solution evaporates quickly at high temperatures, performing PCR on a nanoliter scale is difficult. As a result, the most critical issue in dPCR is water evaporation control during heating, as evaporation will change the dPCR component concentration, resulting in a dPCR failure. Surface absorption of dPCR components such as polymerase is another issue in ultra-small volume dPCR because the decreased volume induces a very high surface-to-volume ratio. Until now, the majority of nanoliter dPCR experiments have been conducted using emulsion droplet technology; however, droplet-based dPCR [12,13,14,15] has inherent drawbacks such as droplet coalescence during heating and a lack of capabilities for high throughput digital PCR results detection.

Static droplets array (SDA) or confined droplets array (CDA) is an alternative regime due to its advantages of the fast generation of a large area droplet array and easy registration for detection, both of which could guarantee a reliable result for biomolecule detection. Men et al. [16] reported a nanoliter scale microfluidic SDA for dPCR based on a PDMS microfluidic chip, and a pressurized elastomeric hydration channel was applied to suppress water evaporation. Quake et al. [17] developed a PDMS microfluidic chip for single-cell multiplex PCR analysis by using Quake valves to seal and isolate the liquid. Sundberg et al. [18] proposed a rotating disk microfluidic dPCR chip made of polyethylene terephthalate (PET). However, when generating independent SDA, these microfluidic digital PCR chips require massive hi-precision peripheral equipment to provide a driving force to assist sample loading and isolation. The sample loading method is complicated, and the time required to generate the microarray is lengthy. Ismagilov et al. [19] proposed a dPCR chip, which changes the position of the reaction chamber and channel by moving two glass chips to realize the addition, mixing, and separation of solutions. Although the slip chip does not require other external equipment, the chip sliding process needs to be operated under a microscope in mineral oil, which is more complicated and inconvenient. Qi Song et al. [20] proposed a negative pressure-driven self-priming liquid-dispensing PDMS microfluidic dPCR chip. However, the microfluidic dPCR chip is composed of a 5-layer structure, and the production of the chip is more complicated.

We designed a novel silicon-based fast-generated open well SDA chip and a rapid dPCR detection platform with a pressurized elaborate water evaporation control system in this paper. The dPCR platform is made up of three major components: a dPCR chip, a data acquisition system, and a pressurized thermal cycler system. We used photolithography and dry etching processing technology to fabricate 2704 independent microarrays on a 15 mm × 15 mm silicon substrate chip. The volume of each micro-reaction chamber was about 0.785 nL. The chip is subjected to a series of hydrophilic treatments during the preparation process, resulting in the surface of the microwells with high-hydrophilic properties. A direct scraping method was used to introduce samples into the nanoliter open well array (OWA) using the surface tension of the sample, and then a glass sheet with oil dripping was covered on the silicon chip. The process for preparing the oil-covered SDA has the advantages of simplicity, high efficiency, high throughput, short sampling time, and no need for additional precision instruments. This platform can monitor each chamber fluorescence using a camera with a wide field of view, allowing for the detection of PCR to collect fluorescence in the bands of 490–510 nm in real-time. We also designed and integrated a pressurized microheater-based thermal cycler for a fast and robust PCR realization to effectively suppress water evaporation in the nanoliter dPCR process.

## 2. Materials and Methods

### 2.1. Open Well SDA Chip

**Figure 1:**
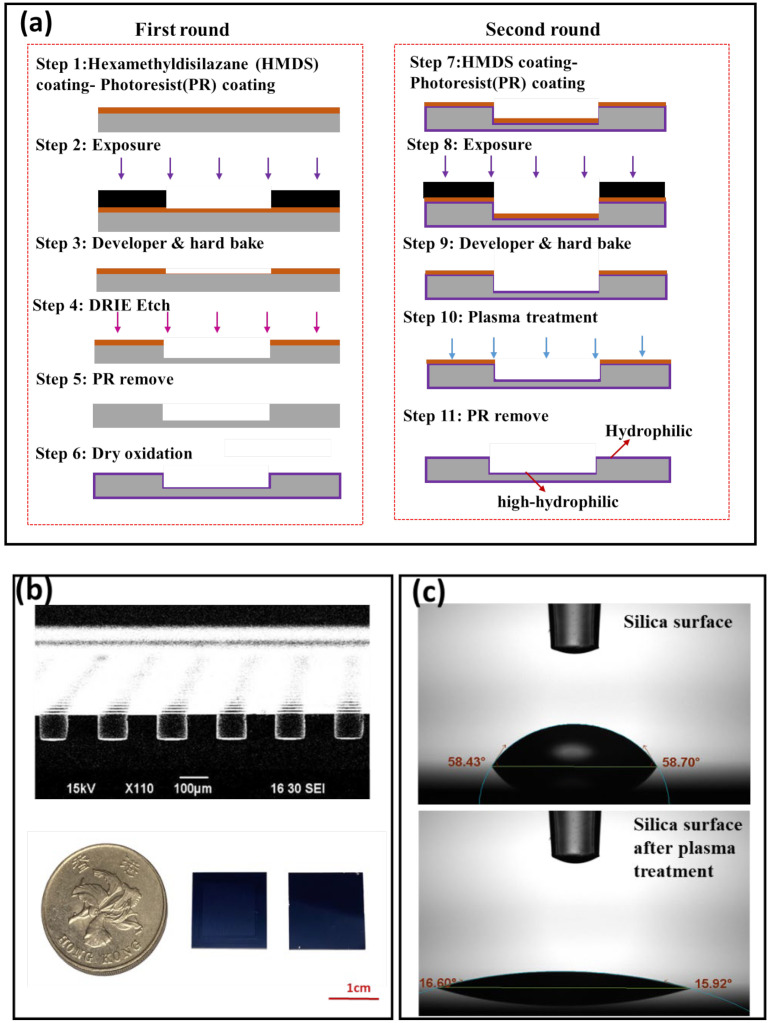
(**a**) The fabrication process of the SDA dPCR chip. (**b**) The upper part of the figure is the SEM image of the etched cross-section of the chip, and the lower part of the figure is the image of the dPCR chip. (**c**) Contact angle on silica surface and after plasma treatment.

The digital PCR chips were designed and fabricated using soft photolithography and dry etching processing technology. The chip contains 2704 independent uniform chambers on a 15 mm × 15 mm silicon substrate chip. The volume of each reaction chamber is 0.785 nL. Figure 1a shows detailed information on the fabrication process flow of the nanoliter OWA. We chose the deep reactive ion etching (DRIE) process to produce the nanoliter well. The DRIE process has the advantages of the formation of microwells with a very vertical profile under a Bosch process. Figure 1b shows the SEM image of the etched cross-section of the chip. The depth of the microwells is about 100 μm, and its depth and size are very uniform, so the volume of each microwell is relatively consistent. The chip was subjected to plasma treatments during the preparation stage, and Figure 1c depicts the contact angle of the silicon surface before and after the plasma treatment. We found that the contact angle on the silica surface was significantly reduced after plasma treatment.

### 2.2. Real-Time Fluorescence Monitoring System

**Figure 2:**
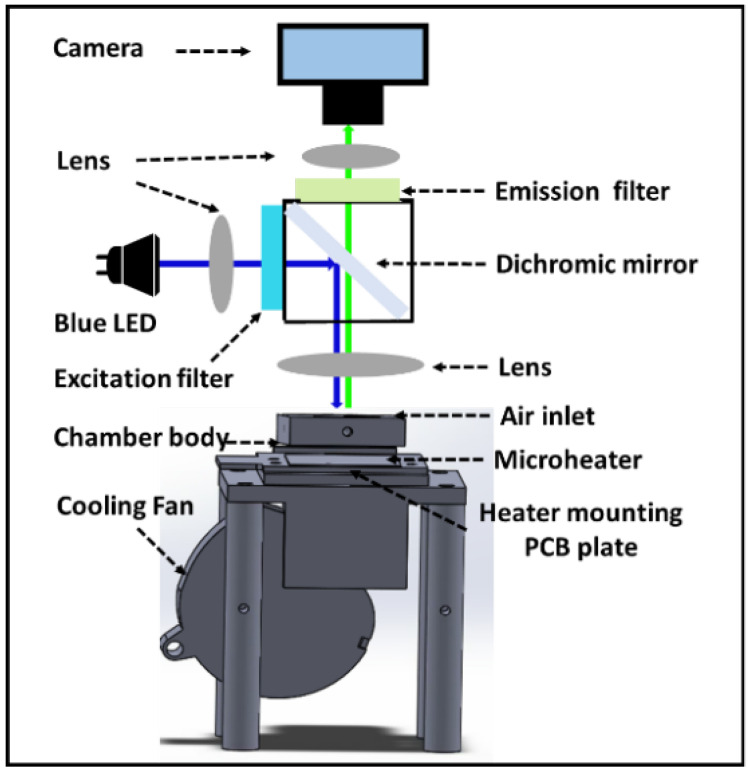
Schematic diagram of detection light path and high-pressure thermal cycling device.

The optical inspection system included a blue-color LED light source with a 472 nm peak wavelength (Ledguhon, Guangzhou, China), a set of filters, a set of lenses, and a CMOS camera (acA5472-17ucMED, Basler, Ahrensburg, Germany). Figure 2 shows the schematic diagram of the optical path detection system. Filters include a dichroic mirror (ZT473/532/633rpc-UF1, CHROMA, Vermont, USA), excitation filter (ET Dapi/FITC/Cy3/Cy5m, CHROMA, Vermont, USA), and emission filter (ET Dapi/FITC/Cy3/Cy5x, CHROMA, Vermont, USA). The dichroic mirror is placed in the light path at 45° to selectively reflect light with wavelengths of 450–490 nm while transmitting light with wavelengths of 490–510. The blue excitation light emitted by the LED is reflected by the dichroic mirror and penetrates the glass layer of the PCR chip to reach the PCR reaction solution. The fluorescence (490–510 nm) emitted when excited by blue light can pass through the glass layer, lens, dichroic mirror, emission filter, and finally reach the CMOS camera. The emission filter can effectively prevent the excitation light reflected from the dPCR chip or the excitation light transmitted through the dichroic mirror from reaching the CMOS camera.

### 2.3. Design and Configuration of the Pressurized Thermal Cycler (PTC)

The first draft of our high-pressure microheater device was created using the commercial 3D design software Solidwork (Dassault Systemes S.A, Vélizy-Villacoublay, France). Figure 2 depicts the integration of the microheater and high-pressure chamber as the key design of the high-pressure microheater device. The chamber body was placed on top of the microheater and sealed with an O-ring. The chamber cover was equipped with a glass window for optical detection and was secured with a lock. A through-hole was cut on the top of the chamber body for air injection to create the high-pressure environment in the dPCR reaction. In the dPCR reaction, air could be injected into the reaction chamber and held in place with the help of an external check valve. In our test, the maximum pressure in the reaction chamber could be up to 0.50 MPa, and in fact, we chose 0.4 MPa in the dPCR reaction, which could provide effective air-sealing and anti-evaporation.

**Figure 3:**
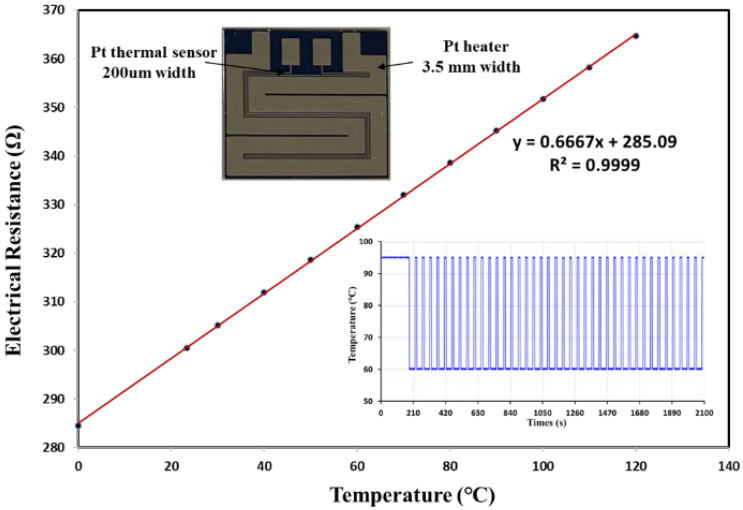
The relationship between Pt sensor resistance and temperature where the correlation coefficient $\left(r=0.9999\right)\;$and PCR temperature pattern of thermal cycling.

A silicon-based microheater with high thermal conductivity was designed and manufactured in our previous work to achieve rapid thermal cycling in PCR [21]. Electrical insulation was provided by 1 mm thermal oxide prior to electrode patterning. Then, using the lift-off sputtering technique, a 2500 Å thin film of platinum (Pt) was deposited on the substrate. The platinum resistivity in our process was approximately 1.06 × 10^−7^ Ω·m, and with the designed pattern, the resistance of the heating terminal and temperature sensor terminal was 16.8 Ω and 300.5 Ω at room temperature, respectively. The resistance of the Pt sensor increased as the temperature of the microheater increased. Platinum, as a noble metal, has excellent linearity between resistance and temperature over a wide temperature range. Figure 3 shows good linearity between Pt sensor resistance and temperature where the correlation coefficient (r) is 0.9999.

### 2.4. Sample Loading and Sealing

**Figure 4:**
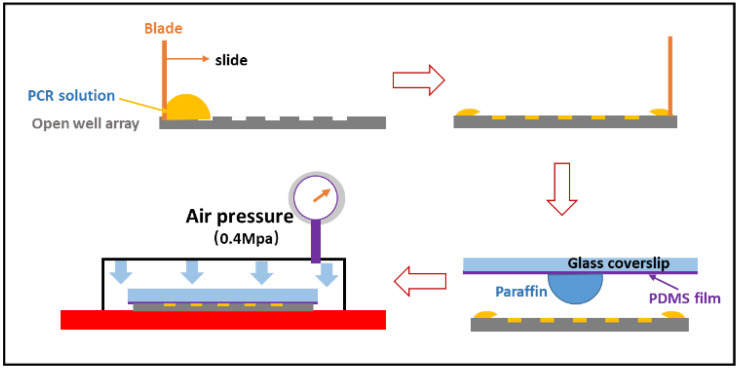
The diagram of sample loading and sealing for the SDA dPCR chip.

A sandwich method [22] was used to trap single molecules in a microfabricated nanoliter well. In this case, we improved nanoliter SDA production to meet the PCR requirement. As shown in Figure 4, we loaded the sample into the nanoliter well using the direct scraping method. The sample was first deposited on a silica substrate before being scraped into the well with clean polydimethylsiloxane (PDMS). Following that, we dropped a suspended paraffin oil on the cover glass with a thin layer of PDMS, and the coverslip was slowly moving down to approach the OWA. The sample could be completely squeezed out of the interface between the coverslip and OWA, leaving only the sample trapped in the nanoliter well.

### 2.5. Digital PCR

We chose the hepatitis B virus (HBV) plasmid as the target DNA to use on the digital PCR chip to test the quantitative detection of the designed digital PCR chip. A 5 μL reaction system was used in the reaction system. Each 5 μL PCR solution contains 2.5 μL 2x TaqMan gene expression master mix (Thermofisher Scientific, Waltham, MA, USA), 0.4 μL probe primer mixture, 1 μL template solution, and 1.1 μL nuclease-free water.

The HBV plasmid template was diluted with nuclease-free water. Before adding the reagents to the chip, they were thoroughly mixed. In the reaction cycle of the dPCR experiment, preheating was first performed at 95 °C for 3 min, followed by 40 cycles of reaction at 95 °C for 15 s and 60 °C for 45 s.

## 3. Results and Discussion

### 3.1. The Uniformity of Test Solution

**Figure 5:**
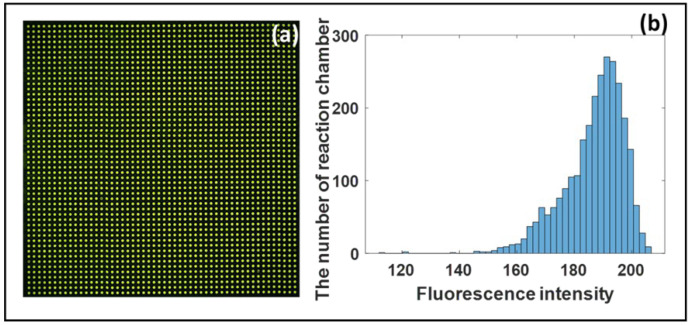
(**a**) Fluorescent images of the solution distribution in the microfluidic chip. (**b**) Histograms of the fluorescence intensity of every reaction chamber.

The principle of the digital PCR of the microfluidic chip is based on the fact that the sample to be tested can be randomly allocated to each reaction chamber, so it is essential to ensure the random allocation of the sample to be tested. The volume of the solution to be tested distributed in each reaction chamber is the same in theory if the reaction chambers are evenly distributed, as is the probability of the sample molecules being dispersed in each reaction chamber. In this experiment, we used fluorescein amidite as a dye to test the solution distribution. Figure 5a shows the solution distribution in the microfluidic chip after loading the solution. While Figure 5b shows the histograms showing the fluorescence intensity of every reaction chamber. As shown in Figure 5b, we found that before reaction, the average fluorescent intensity of all reactors was about 186.81, and the relative standard deviation was about 5.64%, so the fluorescence intensity of each chamber was very uniform, and the sample volume of each reactor was basically the same.

### 3.2. Analysis of Chip Anti-Evaporation Effect

**Figure 6:**
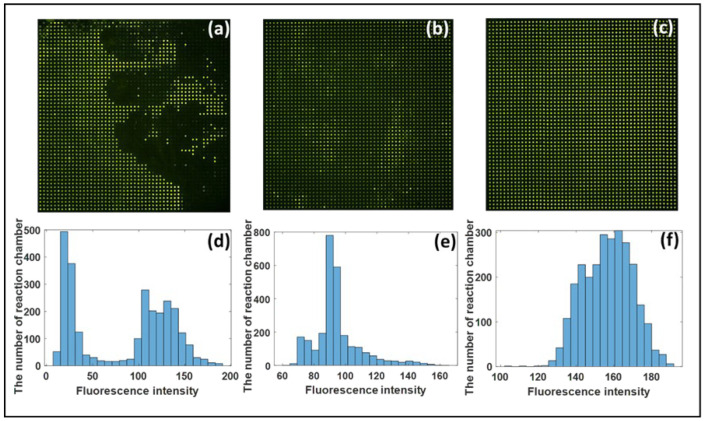
Anti-evaporation effect tests using fluorescein amidite for dPCR chips under different pressures: (**a**) Under standard atmospheric pressure; (**b**) under two atmospheric pressures; (**c**) under four atmospheric pressures. Histograms of the fluorescence intensity of every reaction chamber: (**d**) Under standard atmospheric pressure; (**e**) under two atmospheric pressures; (**f**) under four atmospheric pressures.

It is necessary to heat and maintain a high temperature of 95 °C for an extended period of time during the digital PCR reaction process. The liquid in the reaction chamber is easily evaporated. A large amount of reagent evaporation will seriously affect the accuracy of the experimental results if no anti-evaporation measures are taken. As a result, it is critical to develop a simple anti-evaporation method. To simulate the real-time PCR reaction, we used the fluorescein amidite solution to conduct the experiments. The chip was heated according to the PCR reaction program, and the fluorescence image was captured after the heating was complete. Figure 6 depicts the image of the end result. As shown in Figure 6d–f, under standard atmospheric pressure, two atmospheric pressure, and four atmospheric pressure, the average fluorescence intensities of all chambers were about 82.93, 94.45, and 156.75, and the relative standard deviations were approximately 62.4%, 15.7%, and 8.15%. Figure 6a,d shows that the liquid evaporated rapidly under one atmospheric pressure, and the fluorescence intensity between the wells was greatly uneven. The figure shows that the liquid evaporated rapidly under one atmospheric pressure. Although the difference in the fluorescence intensity between the wells was not significant, the liquid’s anti-evaporation effect improved under two atmospheric pressures. Still, the average fluorescence intensity had nearly dropped by half. There was almost no evaporation under four atmospheric pressures. The liquid’s brightness between the wells was very uniform, there was no crosstalk between the wells, and the effect was excellent.

### 3.3. Digital PCR Analysis

**Figure 7:**
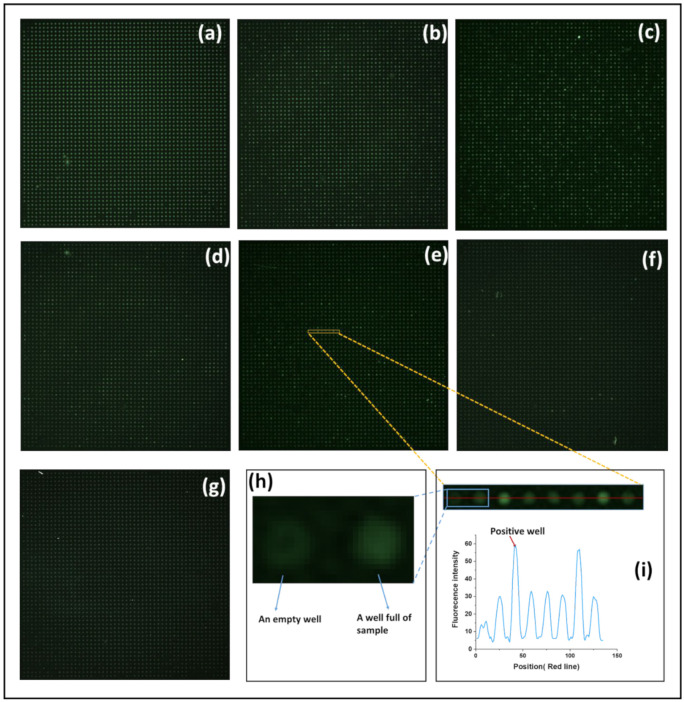
Fluorescence images of the detection results of the digital PCR chip to detect different concentrations of HBV plasmids. The HBV plasmid dilution concentrations were (**a**) 10^−1^; (**b**) 10^−2^; (**c**) 5 × 10^−3^; (**d**) 10^−3^; (**e**) 5 × 10^−4^; (**f**) 10^−4^; (**g**) no template control (NTC). (**h**) The image of an empty well and a well full of the sample. (**i**) A region of interest with eight chambers and the fluorescence intensity distribution in the red line position.

Serial dilutions of HBV plasmid solutions of 10^−1^, 10^−2^, 5 × 10^−3^, 10^−3^, 5 × 10^−4^, and 10^−4^ were prepared. We repeated the experiments for each dilution of HBV plasmid solution three times. Fluorescence images of the detection results of the digital PCR chip to detect different concentrations of HBV plasmids are shown in Figure 7. We found that as the dilution factor of the sample decreased, the number of positive chambers became less.

The distribution of the target molecule on the chip was subjected to Poisson distribution. We calculated the number of DNA molecules in the sample based on the number of bright spots in the collected fluorescence image and the total number of wells. The number of molecules $\left(k\right)$ in the chamber varied, and the fluorescent well contained at least one target molecule, allowing us to derive Equation (1) [20,23].

(1) $$f=P(k>0,\lambda )=1-P\left(k=0,\lambda \right)=1-e^{-\lambda },$$

where λ is the average number of molecules in each chamber, so it can be expressed as $\;\lambda =M/N_{T}$; M is the number of target molecules in the chip; $N_{T}$ is the total number of partitions; $V_{P}$ is the average partition volume; V is the volume of PCR reaction solution; $V_{s}$ is the volume of sample; $X_{dil}$ is the dilution factor; and $C_{0\;}$is the initial concentration of the sample. The number of the positive wells ($N_{P}$) is given as:

(2) $$N_{P}=\left(1-e^{-\lambda }\right)N_{T},$$

Then,

(3) $$\lambda =\ln\left(\frac{N_{T}}{N_{T}-N_{P}}\right)=-\ln\left(1-\frac{N_{P}}{N_{T}}\right)=-\ln\left(1-f\right),$$

Next,

(4) $$M=\frac{C_{0}X_{dil}V_{P}N_{T}V_{S}}{V},$$

(5) $$\lambda =\frac{M}{N_{T}}=\frac{C_{0}X_{dil}V_{P}V_{S}}{V}=1.57\times 10^{-4}C_{0}X_{dil},$$

So,

(6) $$-\ln\left(1-f\right)=1.57\times 10^{-4}C_{0}X_{dil},$$

**Figure 8:**
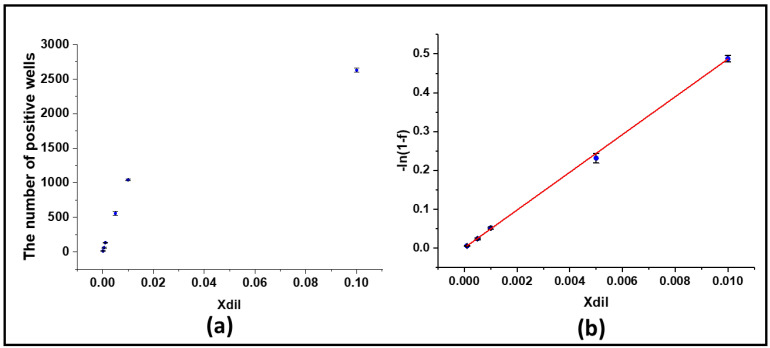
(**a**) Experimental positive wells as a function of dilution factor from 10^−4^ to 10^−1^ (*n* = 3 for the experimental measurement of positive wells). (**b**) Regression fit of digital PCR reaction results. The linear expression of Poisson distribution, where the slope is $1.57\times 10^{-4}C_{0}$.

The linear curve between $-\ln\left(1-f\right)$ and $X_{dil}$ is shown in Figure 8b, where $R^{2}>0.99$. It can be seen from the results that there was good linearity, and the slope was $1.57\times 10^{-4}C_{0}$. From Figure 8b, we can tell that the linear relationship is $y=\left(48.63\pm 0.6151\right)x+0.00004\;(R^{2}>0.99)$, so the initial concentration of sample $C_{0}$ could be calculated as $\left(3.10\times 10^{5}\pm 3918\right)$ copies/μL. The known sample concentration is $3.13\times 10^{5}$ copies/μL, so the calculated value of the experimental result matched the actual value very well.

### 3.4. Real-Time PCR Validation

**Figure 9:**
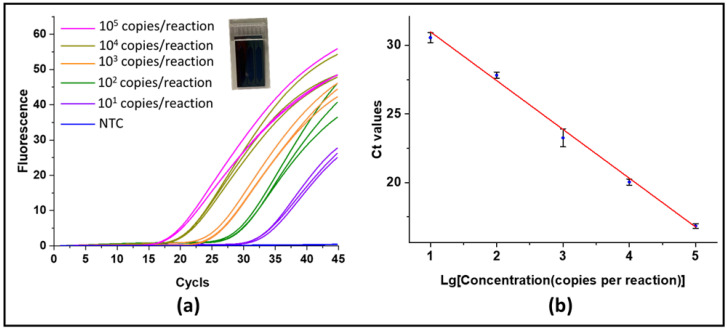
(**a**) Real-time PCR amplification results with three-channel silicon-based chips on digital PCR platform. The HBV plasmid concentrations were $10^{1},\;10^{2},\;10^{3},\;10^{4},\;$and $10^{5}$ copies per reaction, respectively, and the NTC group is not a template control group. (**b**) Standard curve for HBV plasmid quantification.

In this experiment, we conducted real-time PCR reactions using silicon-based three-channel microchips with each volume of about 16 μL for a standard test. We used gradient dilutions of HBV plasmids with concentrations of 3.13 copies/μL to $3.13\times 10^{4}$ copies/μL as DNA templates for PCR reactions. Each 25 μL PCR solution system contained 12.5 μL 2× TaqMan gene expression master mix (Thermofisher Scientific, Massachusetts, USA), 2 μL probe primer mixture, 5 μL template solution, and 5.5 μL nuclease-free water. Therefore, each channel obtained 10 to $10^{5}$ DNA copies per reaction. In the reaction cycle of the PCR experiment, preheating was first performed at 95 °C for 3 min. Subsequently, 45 cycles of reaction amplification were performed at 95 °C for 15 s and at 60 °C for 45 s. Figure 9a presents the amplification results of the PCR reaction with different concentrations, and Figure 9b shows a standard curve of HBV plasmids. As shown in Figure 9a, we found that the Ct value increased as the concentration of DNA template decreased. The averages of the Ct values corresponding to the template concentrations with $10^{1},\;10^{2},\;10^{3},\;10^{4},\;$and $10^{5}$ copies per reaction were about 30.56, 27.83, 23.26, 20.04, and 16.84, respectively. From Figure 9b, we show that the linear relationship is $y=\left(-3.56\pm 0.1138\right)x+\left(34.55\pm 0.4576\right)\;(R^{2}>0.99)$. Therefore, we can use the Ct value to quantify the concentration of DNA. Then, we used 0.1 dilutions of HBV plasmid solution to conduct the real-time PCR reaction, and the Ct value was 16.97. The result of 0.1 dilutions of HBV plasmid calculated according to the standard curve was $2.71\times 10^{4}$ copies/μL, which is consistent with the result of digital PCR. Although real-time PCR can also achieve quantification, it needs a standard curve, and its deviation from the actual concentration is greater than that of digital PCR.

## 4. Conclusions

We created a fast digital PCR platform with fluorescence monitoring in this paper as well as a very simple method to create a large number of nanoliter droplet arrays. To quickly realize thermal cycling in the PCR reaction, a digital PCR chip was made of a silicon substrate with good thermal conductivity. There was no special equipment required for sample loading, and sample loading only took a few seconds. We used gradient dilutions of HBV plasmids as the target DNA and selected plasmids with the dilution scale of 10^−1^, 10^−2^, 5 × 10^−3^, 10^−3^, 5 × 10^−4^, and 10^−4^ as DNA templates for digital PCR reaction. The statistical results showed that the digital PCR chip could realize the absolute quantitative detection of DNA molecules. The upper limit of the detection line and the sensitivity could be increased on the platform by increasing the number of wells on the chip or reducing the volume of a single chamber. Each chip was 15 mm × 15 mm in size, which was only the size of a slide, and it was simple to load samples. As a result, it is ideal for a variety of flexible and broad applications in on-site real-time detection.
